# Machiavellianism, Relationship Satisfaction, and Romantic Relationship Quality

**DOI:** 10.5964/ejop.v13i3.1217

**Published:** 2017-08-31

**Authors:** Gayle Brewer, Loren Abell

**Affiliations:** a School of Psychology, University of Liverpool, Liverpool, United Kingdom; b Nottingham Trent University, Nottingham, United Kingdom; Webster University Geneva, Geneva, Switzerland; University of Liverpool, Liverpool, United Kingdom

**Keywords:** Machiavellianism, relationships, satisfaction, trust, commitment, control, emotional abuse

## Abstract

Machiavellianism is characterised by a manipulative interpersonal style, willingness to exploit others, and a preference for emotionally detached relationships. The present studies investigate the extent to which Machiavellianism influences relationship satisfaction and romantic relationship quality. In Study 1, 194 heterosexual partnered women completed Machiavellianism and Relationship Satisfaction measures. Women with higher levels of Machiavellianism reported lower levels of relationship satisfaction. In Study 2, 132 heterosexual partnered women completed Machiavellianism, Trust, Commitment, Control, and Emotional Abuse scales. Women with higher levels of Machiavellianism perceived their partners to be less dependable, reported less faith in their partners, and were less willing to persist with the relationship than those with low levels of Machiavellianism. With regards to negative behavior, Machiavellianism predicted each form of control and emotional abuse investigated, such that those with high levels of Machiavellianism were more likely to engage in controlling behavior and emotional abuse. Findings have important implications for the prediction of romantic relationship quality and in particular for negative behavior such as control and abuse.

Machiavellianism is characterised by cynicism, manipulation, and a willingness to exploit others (Christie & Geis, 1970). Previous research has demonstrated the manner in which Machiavellianism influences sexual and romantic relationships (Brewer & Abell, 2015a). In particular, men and women with high levels of Machiavellianism prefer emotionally detached relationships and are often reluctant to commit (Ali & Chamorro-Premuzic, 2010). Research has therefore primarily focused on Machiavellianism in the context of short-term sexual rather than long-term committed relationships. Machiavellian men and women do however enter long-term relationships, and these provide valuable opportunities for manipulation and exploitation of the partner (Brewer & Abell, 2015b). The present studies investigate the nature of these relationships. In particular we examine the extent to which Machiavellianism influences women’s relationship satisfaction (Study 1), and specific positive and negative aspects of romantic relationship quality (Study 2).

## Satisfaction

Relationship satisfaction refers to a subjective evaluation of the relationship, involving both positive and negative feelings towards a partner and overall attraction to the relationship (Rusbult & Buunk, 1993). Satisfaction is associated with a range of important relationship outcomes including dissolution (Gottman & Levenson, 1992) and displays considerable individual variation. For example low neuroticism, high agreeableness, high conscientiousness, and high extraversion each predict greater relationship satisfaction (Malouff, Thorsteinsson, Schutte, Bhullar, & Rooke, 2010). Machiavellianism is associated with lower levels of agreeableness and conscientiousness (Austin, Farrelly, Black, & Moore, 2007). Adults with higher Machiavellianism scores have negative representations of others (Ináncsi, Láng, & Bereczkei, 2015) and view others with emotional detachment, distrust, and suspicion (Christie & Geis, 1970). This broad negative view of others coupled with their lack of connection to their own and others feelings (Wastell & Booth, 2003) may result in lower relationship satisfaction. Individuals higher on Machiavellianism may not view relationships themselves as satisfying but engage in relationships to adhere to social norms or in order to manipulate relationship partners (Ináncsi, Láng, & Bereczkei, 2015).

## Trust and Commitment

Trust and commitment are important features of established romantic relationships (Gere & MacDonald, 2013). Trust refers to the expectation of positive reward and partner responsiveness and exerts a substantial impact on relationship quality (Givertz, Woszidlo, Segrin, & Knutson, 2013). In particular, those with higher levels of trust in a partner display resilience to partner criticism (Murray, Lupien, & Seery, 2012), a positive memory bias for previous behavior (Luchies et al., 2013), greater intimacy, and lower partner avoidance behaviors (Wieselquist, Rusbult, Foster, & Agnew, 1999). Commitment refers to a subjective state of dependence on another individual which motivates behavior intended to maintain and strengthen the relationship (Kelley et al., 2003). It is associated with a range of positive relationship behavior such as willingness to support a partner’s interests (Rusbult, Olsen, Davis, & Hannon, 2004) and reduced attention to alternative partners (Miller, 1997). Relationship outcomes associated with commitment include sexual satisfaction (Sprecher, 2002) and relationship dissolution (Le, Dove, Agnew, Korn, & Mutso, 2010).

Those with high levels of Machiavellianism display cynicism and a lack of faith in humanity (Christie & Geis, 1970), which may lead to low levels of relationship trust. Indeed, Ináncsi, Láng, and Bereczkei (2015) comment that “Machiavellian individuals not only have a negative representation of significant others, but they also tend to seek symbiotic closeness in order to exploit their partners” (p. 139). Furthermore, previous research has indicated that Machiavellianism and the closely related traits of narcissism and psychopathy (Ali & Chamorro-Premuzic, 2010; Foster, Shrira, & Campbell, 2006) are associated with low levels of relationship commitment. These studies did not necessarily require participants to be in a relationship at the time of the study. Hence the present study investigates the relationship between Machiavellianism and commitment in a partnered sample. Based on these findings and the previously documented preference for relationships with low levels of commitment (Jonason, Luevano, & Adams, 2012), those with high levels of Machiavellianism are predicted to report low levels of relationship trust and commitment. These findings are consistent with recent findings indicating that women with high levels of Machiavellianism are more likely to enter relationships to obtain sex than affiliation (Brewer, Abell, & Lyons, 2016) and are more likely to report that their needs could be met by alternate partners (Abell & Brewer, 2016).

## Control and Emotional Abuse

Intimate partner violence impacts on a substantial number of relationships (Garcia-Moreno, Janse, Ellsberg, Heise, & Watts, 2006) and may take the form of physical, psychological, or sexual abuse (Coker, Smith, McKeown, & King, 2000). Whilst research has often focused on the consequences of physical violence, psychological abuse predicts a range of negative outcomes including poor physical and mental health (Straight, Harper, & Arias, 2003; Tiwari et al., 2008). Furthermore, it may be more difficult for victims or professionals to recognise and address psychological compared to physical abuse.

Previous research indicates that psychoticism (a closely related trait) is associated with domestic violence (Holtzworth-Munroe, Meehan, Herron, Rehman, & Stuart, 2003). Furthermore, Machiavellianism is associated with a range of behaviors suggestive of a positive relationship with psychological abuse, including a game-playing style of love (Jonason & Kavanagh, 2010), violence (Pailing, Boon, & Egan, 2014), and aggression (Webster, Gesselman, Crysel, Brunell, & Jonason, 2014). Though few studies have considered the relationship between Machiavellianism and psychological abuse directly, recent research reports that those with high levels of Machiavellianism are more likely to engage in emotional abuse (Carton & Egan, 2017). Control forms a central component of intimate partner violence (Felson & Messner, 2000) and may be adopted prior to or as a substitute to violence (Graham-Kevan & Archer, 2009). Previous research has established that Machiavellianism is associated with social dominance (Hodson, Hogg, & MacInnis, 2009) and a desire to maintain power over others (Paulhus & Williams, 2002) therefore positive relationships between Machiavellianism and partner control and emotional abuse are predicted.

The current studies investigate the extent to which Machiavellianism influences women’s romantic relationship quality. We predict that women with high levels of Machiavellianism will report lower levels of relationship satisfaction (Study 1), lower levels of trust and commitment, and an increased need for relationship control and use of emotional abuse (Study 2).

## Study 1

### Method

#### Participants

Heterosexual women (*N* = 194) aged 16-61 years (*M* = 24.94, *SD* = 8.68) were recruited via online research websites and social networking sites. All participants were in an exclusive romantic relationship at the time of the study (*M* = 3.42 years, *SD* = 5.14 years).

#### Materials and Procedure

Each participant completed the Mach IV (Christie & Geis, 1970) and Relationship Satisfaction Scale (Hendrick, 1988). The *Mach IV* (Christie & Geis, 1970) contains 20 items rated on a seven point scale (1 = *strongly disagree* to 7 = *strongly agree*). The scale assesses interactions with others, morality, and cynicism. Example items include “Anyone who completely trusts anyone else is asking for trouble” and “Never tell anyone the real reason you did something unless it is useful to do so”. Ten items were reverse scored such that higher scores indicate higher Machiavellianism.

The Relationship Satisfaction Scale (Hendrick, 1988) contains 7 items each answered on a five point scale (1 = *low* to 5 = *high*). Example items include “In general, how satisfied are you with your relationship” and “To what extent has your relationship met your original expectations”. Two items were reverse scored such that higher scores represent higher levels of relationship satisfaction. Cronbach’s alphas in the current study were Machiavellianism: α = .64 and Satisfaction: α = .91.

### Results

Participants completed standardized measures assessing Machiavellianism and Relationship Satisfaction. These were significantly correlated, *r*(181) = -.40, *p* < .01, such that high Machiavellianism scores were associated with low relationship satisfaction. A multiple regression analysis was conducted to investigate the extent to which Machiavellianism predicted relationship satisfaction. The influence of relationship length was also investigated, both as an individual predictor and as a moderator of the relationship between Machiavellianism and relationship satisfaction. To represent the interaction between Machiavellianism and relationship length, these variables were first mean centered and multiplied together. Both individual predictors and the interaction term were then entered into a simultaneous regression model. The model significantly predicted relationship satisfaction (*R*^2^ = .15, *F*(3,164) = 9.69, *p* < .001) and Machiavellianism was a significant individual predictor (*β* = -.36, *t* = -5.04, *p* < .001), such that higher levels of Machiavellianism were associated with lower relationship satisfaction. Relationship length was not a significant individual predictor and did not moderate the influence of Machiavellianism on relationship satisfaction.

## Study 2

### Method

#### Participants

Heterosexual women (*N* = 132) aged 18-50 years (*M* = 25.70, *SD* = 8.58) were recruited online via research websites and social networking sites. All participants were in a romantic relationship at the time of the study (*M* = 3.65 years, *SD* = 3.96 years).

#### Materials and Procedure

Each participant completed initial demographic questions followed by a series of standardised measures including the Mach IV (Christie & Geis, 1970), Trust in Close Personal Relationships Scale (Rempel, Holmes, & Zanna, 1985), Commitment Scale (Rusbult, Kumashiro, Kubacka, & Finkel, 2009), Interpersonal Violent Control Scale (Bledsoe & Sar, 2011) and the Multidimensional Measure of Emotional Abuse (Murphy & Hoover, 1999).

The *Mach IV* (Christie & Geis, 1970) contains 20 items rated on a seven point scale (1 = *strongly disagree* to 7 = *strongly agree*). As outlined previously, the scale assesses interactions with others, morality, and cynicism. Example items include “The best way to handle people is to tell them what they want to hear” and “It is wise to flatter important people”.

The Trust in Close Personal Relationships Scale (Rempel, Holmes, & Zanna, 1985) is a 17 item measure of trust in a relationship partner. Participants report the extent to which they agree or disagree with a series of statements such as “I can rely on my partner to keep the promises he/she makes to me” from -3 (*strongly disagree*) to 3 (*strongly agree*). The measure contains three subscales: predictability (5 items); dependability (5 items); and faith (7 items).

The Commitment Scale (Rusbult et al., 2009) is a 15 item measure of relationship commitment. Participants indicate the extent to which they agree or disagree with a series of statements on a nine-point scale from 0 (*do not agree at all*) to 8 (*agree completely*). Example statements include “I feel completely attached to my partner and our relationship”. The measure contains three subscales: intent to persist (5 items); attachment (5 items); and long-term orientation (5 items).

The Intimate Partner Violence Control Scale (Bledsoe & Sar, 2011) is a 16 item measure of desired control. The measure contains three subscales: control through surveillance and threats (e.g. wishing to keep track of a partner, 6 items); control over everyday routines and decision making (e.g. wishing to control how a partner spends their day, 5 items); and control over autonomous behavior (e.g. wishing that the partner would terminate their job, 5 items). Participants respond to statements such as “I wish I had more control of how my partner spends the day” on a five-point scale from 1 (*never*) to 5 (*very often*). In the present study one item (“I wish sometimes that I could take the children away from my partner to get him/her to go along with things”) was removed from the control through surveillance and threats subscale, as not all participants were expected to be parents.

The Multidimensional Measure of Emotional Abuse (Murphy & Hoover, 1999) is a 28 item measure of psychological abuse. Participants rate the frequency of abuse during the previous six months on an eight-point scale from 1 (*once*) to 6 (*more than 20 times*), with ‘*never in the past six months but it has happened before’* (7) and ‘*this has never happened’* (0) options also provided. Participants responded to all items (e.g. “Belittled the other person in front of other people”) as a perpetrator. The measure contains four subscales, each containing 7 items. The subscales were: restrictive engulfment (e.g. complaining that a partner spends too much time with friends); denigration (e.g. calling a partner a failure or worthless); hostile withdrawal (e.g. refusing to acknowledge or discuss a problem); and dominance / intimidation (e.g. threatening a partner or destroying their belongings).

Higher scores indicate greater levels of Machiavellianism and each aspect of relationship quality investigated. Cronbach’s alphas were acceptable in the current study: Machiavellianism (α = .72); Dependability (α = .77); Faith (α = .93); Intent to Persist (α = .92); Attachment (α = .72); Long-Term Orientation (α = 85); Surveillance and Threats (α = .79); Everyday Routines and Decision Making (α = .74); Autonomous Behavior (α = .73); Restrictive Engulfment (α = .86); Denigration (α = .89); Hostile Withdrawal (α = .90); Dominance / Intimidation (α = .83) investigated. The Cronbach’s alpha for Predictability was unacceptably low (α =.32) but increased to α =.68 following the removal of item 8. Therefore the modified variable was used for subsequent analyses.

### Results

Participants completed standardized measures assessing Machiavellianism, Trust, Commitment, Control, and Emotional Abuse. Descriptive statistics and correlations for these variables are displayed in Table 1. Multiple regression analyses were conducted to investigate the extent to which Machiavellianism predicted relationship quality. The influence of relationship length was also investigated, both as an individual predictor and as a moderator of the relationship between Machiavellianism and relationship quality. To represent the interaction between Machiavellianism and relationship length, these variables were first mean centered and multiplied together. Both predictors and the interaction term were then entered into a simultaneous regression model.

**Table 1:** Descriptive Statistics and Correlations for Machiavellianism, Trust, Commitment, Control, and Emotional Abuse

	MA	PRE	DEP	FAI	INT	ATT	LON	SUR	EVE	AUT	RES	DEN	HOS	DOM
**MA**		.24**	-.26**	-.35**	-.38**	-.14*	-.22*	.39**	.33**	.41**	.38**	.35**	.45**	.31**
**PRE**			-.55**	-.62**	-.39**	-.36**	-.49**	.58**	.43**	.41**	.38**	.33**	.42**	.273**
**DEP**				.73**	.45**	.41**	.41**	-.66**	-.40**	-.33**	-.46**	-.34**	-.42**	-.21*
**FAI**					.63**	.49**	.60**	-.62**	-.47**	-.36**	-.41**	-.35**	-.50**	-.29**
**INT**						.71**	.81**	-.43**	-.28**	-.36**	-.24*	-.35**	-.43**	-.35**
**ATT**							.60**	-.32**	-.12	-.26**	-.11	-.24*	-.28**	-.21*
**LON**								-.39**	-.24*	-.27**	-.19	-.34**	-.36**	-.35**
**SUR**									.61**	.66**	.70**	.58**	.57**	.45**
**EVE**										.54**	.61**	.30**	.38**	.24*
**AUT**											.49**	.54**	.48**	.52**
**RES**												.62**	.55**	.47**
**DEN**													.67**	.79**
**HOS**														.65**
**DOM**														
***M***	65.37	-4.05	6.32	12.46	31.19	27.66	30.93	9.18	8.93	5.36	7.30	3.33	9.08	2.75
***SD***	13.02	5.41	6.77	9.51	8.56	6.99	7.99	3.93	3.81	2.31	7.61	6.15	8.85	6.80

*Note.* MA = Machiavellianism; PRE = Predicatability; DEP = Dependability; FAI = Faith; INT = Intent to Persist; ATT = Attachment; LON = Long-Term Orientation; SUR = Surveillance and Threats; EVE = Everyday Routines and Decision Making; AUT = Autonomous Behavior; RES = Restrictive Engulfment; DEN = Denigration; HOS = Hostile Withdrawal; DOM = Dominance / Intimidation.

*p < .05. **p < .01.

The model significantly predicted the dependability (*R*^2^ = .10, *F*(3,111) = 3.87, *p* = .011) and faith (*R*^2^ = .17, *F*(3,109) = 7.30, *p* < .001) components of relationship trust but not predictability. Machiavellianism was a significant individual predictor of dependability (*β* = -.25, *t* = -2.74, *p* = .007) and faith (*β* = -.35, *t* = -4.01, *p* < .001) such that those with higher levels of Machiavellianism perceived their partners to be less dependable and reported less faith in their partners than those with low levels of Machiavellianism. Relationship length was also a significant individual predictor of faith (*β* = -.20, *t* = -2.31, *p* = .023), such that those in longer term relationships reported less faith in their partners. The model also predicted the intent to persist (*R*^2^ = .15, *F*(3,103) = 5.90, *p* = .001), but not long-term orientation (*R*^2^ = .06, *F*(3,103) = 2.17, *p* = .100), or attachment (*R*^2^ = .03, *F*(3,103) = 1.13, *p* = .340) components of relationship commitment. Machiavellianism was a significant individual predictor of intent to persist (*β* = -.38, *t* = -4.17, *p* < .001), such that women with high levels of Machiavellianism were less willing to persist with the relationship than those with low levels of Machiavellianism. Relationship length was not a significant individual predictor of trust or commitment (with the exception of faith) and did not moderate the influence of Machiavellianism on these variables.

The model predicted the surveillance and threats (*R*^2^ = .28, *F*(3,91) = 11.59, *p* < .001), everyday routines and decision making (*R*^2^ = .13, *F*(3,93) = 4.80, *p* < .001), and autonomous behavior (*R*^2^ = .17, *F*(3,90) = 6.25, *p* = .001) components of relationship control. Machiavellianism was a significant individual predictor of surveillance and threats (*β* = .38, *t* = 4.24, *p* < .001), everyday routines and decision making (*β* = .32, *t* = 3.33, *p* = .001), and autonomous behavior (*β* = .41, *t* = 4.23, *p* < .001), such that those with high levels of Machiavellianism were more likely to engage in each form of controlling behavior. Relationship length was also a significant individual predictor of surveillance and threats (*β* = .35, *t* = 3.92, *p* < .001), such that women in longer term relationships were more likely to employ this form of controlling behavior.

The model predicted the restrictive engulfment (*R*^2^ = .17, *F*(3,90) = 6.07, *p* = .001), denigration (*R*^2^ = .14, *F*(3,89) = 4.64, *p* = .005), hostile withdrawal (*R*^2^ = .34, *F*(3,89) = 9.30, *p* < .001), and dominance / intimidation (*R*^2^ = .11, *F*(3,88) = 3.46, *p* = .020) forms of emotional abuse. Machiavellianism was a significant individual predictor of restrictive engulfment (*β* = .36, *t* = 3.72, *p* < .001), denigration (*β* = .35, *t* = 3.54, *p* = .001), hostile withdrawal (*β* = .44, *t* = 4.70, *p* < .001), and dominance / intimidation (*β* = .32, *t* = 3.11, *p* = .003), such that those with high levels of Machiavellianism were more likely to engage in emotional abuse. Relationship length was also a marginally significant individual predictor of hostile withdrawal (*β* = .19, *t* = 2.00, *p* = .049). Relationship length was not a significant individual predictor of controlling behavior (with the exception of surveillance and threats) or emotional abuse (with the marginal exception of hostile withdrawal) and did not moderate the influence of Machiavellianism on controlling behavior or emotional abuse.

## Discussion

The present study demonstrates that women with high levels of Machiavellianism experience low levels of relationship satisfaction, perceive their partner to be less dependable, report less faith in their partners, and are less willing to persist in the relationship. Findings support the assertion that men and women with high levels of Machiavellianism prefer emotionally detached relationships with low levels of commitment (Ali & Chamorro-Premuzic, 2010). The present study also demonstrated that women with high levels of Machiavellianism were more likely to engage in each form of controlling behavior and emotional abuse investigated. These may serve a range of functions (e.g. increase the partner’s dependency, lower the partner’s self-esteem, increase the partner’s insecurity about the relationship, and produce fear or submission) which may make the partner more susceptible to manipulation attempts and / or less likely to confront these women about their behavior.

Previous research indicates that controlling behavior and emotional abuse have a substantial impact on the health and wellbeing of the victim (Coker, Smith, Bethea, King, & McKeown, 2000) with many victims reporting that the emotional abuse exerted a greater impact than the physical abuse. The current findings may therefore assist the identification of those most likely to engage in this behavior. Emotional abuse predicts perpetration of physical aggression and future research should investigate the relationship between Machiavellianism and physical partner violence. As researchers have documented the extent to which mutual aggression within relationships occurs (e.g. Archer, 2000, 2006), research investigating both perpetration and victimization in each partner would be particularly beneficial.

A substantial body of research has documented the manner in which romantic interpersonal styles and relationship preferences develop across the lifespan, particularly with reference to the influence of parent-child relationships (Kelley et al., 2005). In contrast, there is relatively little information available relating to the developmental trajectory of Machiavellianism, though this may also be influenced by parent-child relationships (Abell, Lyons, & Brewer, 2014). Whilst previous research documents the influence of Machiavellianism on children’s behavior towards peers (Abell et al., 2015), research investigating the manner in which Machiavellianism impacts on the formation of romantic relationships in young adolescents is required.

### Limitations and Future Research

The present study was dependent on self-report questionnaire data. Though consistent with research in this area, self-report data are subject to social desirability, random responding, bias interpretation, and recall accuracy. Social desirability may be particularly important for the reporting of negative behavior such as relationship control and emotional abuse, though research indicates a greater willingness to disclose undesirable behavior in online compared to offline studies (Booth-Kewley, Larson, & Miyoshi, 2007). Future research should therefore consider the inclusion of observational data. Whilst it may be difficult to capture infrequent behavior with observational methods, this approach may provide a more realistic account of relationship dynamics such as decision making, negotiation, and conflict resolution (e.g. Pérusse, Boucher, & Fernet, 2012).

Furthermore, the present study investigated the influence of Machiavellianism on one partner’s behavior only. Though there is recent evidence for the existence of assortative mating for Machiavellianism (Smith et al., 2014), women may behave differently when paired with low or high Machiavellian partners. For example, women may be less likely (or less able) to manipulate men with high levels of Machiavellianism due to their cynicism and distrust (Christie & Geis, 1970). Indeed Machiavellianism is associated with the perception that relationship partners engage in emotional manipulation (Abell, Brewer, Qualter, & Austin, 2016). Previous research has also documented important sex differences with regards to relationship behavior (e.g. Archer, 2000), those factors influencing relationship outcomes (Acitelli, 1992), and the manner in which Machiavellianism influences social relationships (Brewer, Abell, & Lyons, 2014). Therefore research should consider both the use of manipulation and those behaviors intended to reduce the threat of exploitation in romantic relationship dyads.

To conclude, Machiavellianism predicted relationship satisfaction and romantic relationship quality. Women with high levels of Machiavellianism reported lower relationship satisfaction, perceived their partners to be less dependable, reported less faith in their partners, and were less willing to persist with the relationship. Women with high levels of Machiavellianism also reported greater use of controlling behavior and emotional abuse directed at their partner. These findings indicate that though women with high levels of Machiavellianism enter long-term relationships, these are more likely to be poor in quality.
